# Physiological Effects and Transcriptomic Analysis of sbGnRH on the Liver in Pompano (*Trachinotus ovatus*)

**DOI:** 10.3389/fendo.2022.869021

**Published:** 2022-05-02

**Authors:** Xilin Ren, Jinlei Liu, Charles Brighton Ndandala, Xiaomeng Li, Yuwen Guo, Guangli Li, Huapu Chen

**Affiliations:** ^1^ Guangdong Research Center on Reproductive Control and Breeding Technology of Indigenous Valuable Fish Species, Guangdong Provincial Engineering Laboratory for Mariculture Organism Breeding. Guangdong Provincial Key Laboratory of Pathogenic Biology and Epidemiology for Aquatic Economic Animals, Fisheries College, Guangdong Ocean University, Zhanjiang, China; ^2^ Southern Marine Science and Engineering Guangdong Laboratory, Zhanjiang, China; ^3^ Guangdong Havwii Agricultural Group Co., Ltd, Zhanjiang, China

**Keywords:** *Trachinotus ovatus*, sbGnRH, transcriptome, liver, physiology

## Abstract

Pompano (*Trachinotus ovatus*) is one of the important economic marine fishes in the south coast of China. At present, the research on the basic biology of pompano is relatively weak, which has seriously affected the development of this economic important fish. The liver is an important digestive and metabolic organ of fish which plays an important regulatory role in its growth and development. It is necessary to clarify the effects of sea bream gonadotropin releasing hormone (sbGnRH) on liver physiology and metabolic enzyme activity. The effects of sbGnRH peptides (10 ng/gbw) on the physiological and biochemical indices and metabolic enzyme activities of pompano liver were studied. It was found that after injection of 10 ng/gbw sbGnRH peptides, the contents of albumin, high-density lipoprotein cholesterol, low-density lipoprotein cholesterol, glucose, creatine kinase, iron, magnesium, aspartate aminotransferase, alanine aminotransferase and creatinine increased, while of cholesterol and calcium contents decreased. The activities of amylase, lipase, pyruvate kinase, acyl CoA oxidase, superoxide dismutase, phospholipid hydroperoxide glutathione peroxidase, catalase, glucose-6-phosphate dehydrogenase, fatty acid synthase and lipoprotein lipase increased, while the activities of malic enzyme, carnitine acyl, carnitine translocation, acetyl CoA carboxylase and malondialdehyde decreased. Three hours after the injection of different concentrations of sbGnRH peptides (0 and 10 ng/gbw), the transcriptome sequences of the two groups of livers were sequenced. After quality control and removal of some low-quality data, clean reads of 21,283,647、19,427,359、21,873,990、21,732,174、23,660,062 and 21,592,338 were obtained respectively. In this study, 99 genes were screened and identified as differentially expressed genes, including 77 up-regulated genes and 22 down-regulated genes. According to the Kyoto Encyclopedia of Genes and Genomes (KEGG) and Gene Ontology (GO) pathway analyses, these pathways and the typical genes involved can be divided into cellular processes, environmental information processing, genetic information processing, diseases, metabolism and organismal systems. The results from this study provide a the oretical basis for studying the effects of sbGnRH on the physiology, biochemistry and metabolic enzyme activities of liver in pompano.

## 1 Introduction

Gonadotropin-releasing hormone (GnRH) is a key hormone produced in hypothalamus and plays an important role in reproductive regulation, regulating the development of vertebrates. In teleosts, mature GnRH is synthesized in hypothalamic neuronal cells, temporarily stored in secretory cells at its nerve endings, and transferred to the pituitary after release. It binds with its receptor in the pituitary and acts on gonadotropin cells to trigger the synthesis and release of gonadotropins (GTHs). Synthetic GTHs reaches the gonad through blood, which can promote steroid hormone production and gametogenesis (1). GnRH produced in the hypothalamus can affect vertebrate reproduction at many levels, such as pituitary and gonad. The most classic pathway is through pituitary mediated signal pathway. GnRH stimulates pituitary synthesis and release of GTHs, and then stimulates a series of downstream reproductive related activities (2–4). It can also adjust the content of local GTHs and the level of sex steroid hormones in the blood by paracrine or autocrine, so as to change the sexual behavior of animals (5–7). GnRH acts through the Gonadotropin releasing hormone receptor (GnRHR) of the 7-transmembrane G protein-coupled receptor (GPCR) superfamily. It indicates that GnRH signal transduction can induce the expression of lh by protein kinase C (PKC) cascade mediated immune regulation mechanism, the expression of fsh by cAMP/protein kinase A (PKA) cascade mediated. Regulation of gonadotropin expression is a key factor in inducing and completing gonadal maturation. Kisspeptin-Kissr can also control hypothalamic GnRH release by activating PLC pathway and intracellular Ca^2+^ mobilization. Kisspeptin also stimulates GnRH neurons through mitogen-activated protein kinase (MAPK), especially ERK1/2, p38 and phosphatidylinositol 3-kinase (PI3K)/Akt activation. The activation of these pathways is also considered necessary for progesterone synthesis and preovulation follicles. This suggests that kisspeptin-Kissr signaling may not be necessary in teleost species, but may be a compensation for the successful development and maturation of normal reproductive axis.

In addition, many studies have also found GnRH or similar peptides in central nervous system, peripheral nerve and sympathetic ganglia by using immunoenzymatic localization, immunohistochemistry, *in situ* hybridization and radioimmunoassay, and GnRH receptor (GnRHR) is also found in these tissues. (8). GnRH is widely distributed in the reproductive system, neural network, endocrine system, immune system and digestive system. By integrating and transmitting the information of each system, they can coordinate and cooperate to achieve the best physiological state (9). In different tissues, GnRH may play different biological functions and maintain the stability of the internal environment. In addition, GnRH can also participate in the regulation of the immune system through paracrine and autocrine. In the digestive system, GnRH participates in metabolic activities as a paracrine regulator; and in cancer cells as autocrine regulators. Among them, sbGnRH mRNA in many fish, such as pompano (*Trachinotus ovatus*) (10), turbot (*Scophthalmus maximus*) (11) and rainbow trout (*Oncorhynchus mykiss*) (12), shows a common tissue expression pattern, and its expression can be detected in different tissues, indicating that sbGnRH has a variety of physiological functions. It can regulate a variety of physiological activities, including reproduction.

The liver is an important digestive and metabolic organ in the growth and development of teleost fish. Studies have shown that the expression of sbGnRH in the fish liver is high, and the exertion of various physiological pathways may be related to the function of sbGnRH, which regulates the metabolism of substances and energy (13, 14). It plays a key role in nutrient utilization, lipid, carbohydrate and protein metabolism, endocrine and immune homeostasis regulation (15). However, there are few studies on the comprehensive evaluation of the effects of sbGnRH on the physiological and metabolic patterns of oval nematode liver, which is of great significance to explore the interaction mechanism between liver and sbGnRH. In this study, the differences of liver physiological indexes of pompano after injection of different concentrations of sbGnRH peptides were detected. Further comparative transcriptome analysis was carried out in order to provide a global view of the role of sbGnRH in liver biology and explore the potential role of sbGnRH in non-reproductive systems. These data are valuable for further elucidating the various physiological functions of sbGnRH in this species.

## 2 Materials and Methods

### 2.1 Fish and Sample Preparation

Adult pompano (*T. ovatus*) (body weight, 300 ± 20 g, body length: 22 ± 3 cm) were purchased from the Dongfeng Market (Zhanjiang, Guangdong, China), female fish were selected according to the observed gonads. The sbGnRH peptide used for this study was purchased from GL Biochem (Shanghai, China). sbGnRH peptides were dissolved in physiological saline. All the fish were anesthetized with 100 mg/L tricaine methane sulfonate (MS-222, Sigma,St. Louis, MO, USA) and dissected. Animal experiments were conducted in accordance with the guidelines approved by the Animal Research and Ethics Committees of Fisheries College of Guangdong Ocean University, Zhanjiang, China.

The dose and sampling time of sbGnRH peptides injection were selected according to our previous research results (10). Female adult fish were intraperitoneally injected with 10 ng/g of sbGnRH peptides according to the average body weight, while the control group was intraperitoneally injected with physiological saline. Each group contained three fishes. Three hours after injection, the selected fish were anesthetized and the liver was collected, frozen in liquid nitrogen and later stored at -80°C.

### 2.2 Measurement of Physiological and Biochemical Indexes and Enzyme Activities of Liver

Liver samples were accurately weighed and homogenized (diluted 1:10) in Phosphate buffered saline (PBS) (Solarbio, Beijing, China) with a tissue crusher. The homogenization was 900 × g centrifuge for 10 min at 4°C and retained the supernatant. The protein content of the homogenate was measured using Folin-phenol reagent (16, 17). The contents of albumin (ALB), cholesterol (CHO), high-density lipoprotein cholesterol (HDLC), low-density lipoprotein cholesterol (LDLC), glucose (GLUC), creatine kinase (CK), iron (IRON), magnesium (MG), calcium (CA), phosphate (PHOS), alkaline phosphatase (ALP), aspartate aminotransferase (AST), alanine aminotransferase (ALT) and creatinine (CREA) were detected by Roche Cobas C311 automatic biochemical analyzer (Shanghai, China). The kits used were Cobas C311 products.

According to the product instructions, amylase (AMS), glucose-6-phosphate dehydrogenase (G-6-PD), malic enzyme (ME), pyruvate kinase (PK), acyl CoA oxidase (ACO), lipase (LPS), fatty acid synthase (FAS), carnitine acyl carnitine translocation (CACT), acetyl CoA carboxylase (ACC), lipoprotein lipase (LPL), superoxide dismutase (SOD), Phospholipid hydroperoxide glutathione peroxidase (GSH-PX), catalase (CAT) and malondialdehyde (MDA) were detected. See **Table S1** for product numbers.

### 2.3 RNA-Seq and Transcriptomic Analysis

Six liver samples (3 replicates per group) were used to prepare a transcriptome (RNA-seq) sequencing libraries by the Biomarker Technologies. The RNA-seq process was performed as follows: 1 μg RNA per sample was used as input material for the RNA sample preparations. Sequencing libraries were generated using NEBNext^®^Ultra™ RNA Library Prep Kit for Illumina^®^ (NEB, USA) following the manufacturer’s recommendations and index codes were added to attribute sequences to each sample. Briefly, mRNA was purified from total RNA using poly-T oligo-attached magnetic beads. Fragmentation was carried out using divalent cations under elevated temperature in NEBNext First Strand Synthesis Reaction Buffer (5X). First strand cDNA was synthesized using random hexamer primer and M-MuLV Reverse Transcriptase. Second strand cDNA synthesis was subsequently performed using DNA Polymerase I and RNase H. Remaining overhangs were converted into blunt ends *via* exonuclease/polymerase activities. After adenylation of 3’ ends of DNA fragments, NEBNext Adaptor with hairpin loop structure was ligated to prepare for hybridization. The library fragments were purified with AMPure XP system (Beckman Coulter, Beverly, USA). Then 3 μl USER Enzyme (NEB, USA) was used with size-selected, adaptor-ligated cDNA at 37°C for 15 min followed by 5 min at 95°C before PCR. PCR was performed with Phusion High-Fidelity DNA polymerase, Universal PCR primers and Index (X) Primer. After which, PCR products were purified (AMPure XP system) and library quality was assessed on the Agilent Bio analyzer 2100 system. After cluster generation, the library preparations were sequenced on an Illumina Hiseq 2000 platform and paired-end reads were generated. Transcriptome assembly was accomplished based on the left.fq and right.fq using Trinity (18) with the min_kmer_cov set to 2 by default and all other parameters set to default.

UniGene sequence was compared with Non-Redundant protein (NR) (19), Swiss prot (20), Clusters of Orthologous Group (COG) (21), Eukaryotic Orthologous Groups (KOG) (22) and evolutionary genealogy of genes: Non-supervised Orthologous Groups (eggNOG 4.5) (23) and Kyoto Encyclopedia of Genes and Genomes (KEGG) (24) database alignment by using diamond (25) software. Kobas (26) was used to obtain the KEGG ontology results of UniGene in KEGG. Interproscan (27) used the database integrated by interpro to analyze the Gene Ontology (GO) (28) ontology results of new genes. After predicting the amino acid sequence of UniGene, HMMER (29) software was used to compare results obtained with Protein Families (Pfam) (30) database to obtain the annotation information of UniGene. Bowtie (31) was used to compare the sequenced reads with the UniGene library. According to the comparison results, the expression level was estimated in combination with RNA-Seq by Expectation-Maximization (RSEM) (32). The expression abundance of the corresponding UniGene was expressed by fragments per kilobase of exon model per million reads mapped (FPKM) value. In the process of differential expression analysis, the recognized and effective Benjamin Hochberg method is used to correct the significance *p*-value obtained from the original hypothesis test. The corrected *p*-value, FDR (false discovery rate), is then used as the key index of differential expression gene screening to reduce the false positive caused by independent statistical hypothesis test on the expression value of a large number of genes (33). In the screening process, *p*-value < 0.01 and the difference multiple FC (fold change) ≥ 1.5 are used as the screening criteria.

### 2.4 Real-Time Quantitative PCR (RT-qPCR) Validation

The expression pattern of differentially expressed genes (DEGs) in RNA-seq analysis was verified by RT-qPCR (34). Total RNA was extracted from liver tissues of the control group and sbGnRH peptides treated group using Trizol according to manufacturer’s instructions (Invitrogen, CA, USA). Each group had 3 repetitions. According to the PrimeScript RT Master Mix Perfect Real -Time Kit (Takara, China) manufacturer’s instructions, each sample has the same amount of RNA for reverse transcription (35, 36). RT-qPCR was performed with the LightCycle 480 system (Roche, Basel, Switzerland) using SYBR Premix Ex Taq II (TaKaRa Bio Inc., Shiga, Japan). Primer pairs used in this study were shown in **Table 1**. The reference gene *β-actin* was used as an internal reference to normalize the mRNA level. The relative gene expression was calculated by 2^−ΔΔCt^ method.

**Table 1:** Primer sequences used in RT-real time quantitative PCR (RT-qPCR).

Gene	Primer name	Primer sequence (5′ -3′)	Purpose
*β-actin*	*β-actin*-F	GAGAGGTTCCGTTGCCCAGAG	Reference gene
	*β-actin*-R	CAGACAGCACAGTGTTGGCGT	Reference gene
*vtg*	*vtg*-F	CTGTGCTGATGGTGCTCTGTTGA	qPCR
	*vtg*-R	CAACAGAGCACCATCAGCACAGA	qPCR
*gpr1*	*gpr1*-F	GTGGTTGCTCAATCTTGCGATGG	qPCR
	*gpr1*-R	ATAATGTGTATCATGGCTGCTGTATGC	qPCR
*egr1*	*egr1*-F	AGAAGCCAGTGGTGGAGCAGAC	qPCR
	*egr1*-R	TGAGGAAGAGGTAGAAGAGGAAGAAGTG	qPCR
*rbm34*	*rbm34*-F	GAAGAAGAGGAAGGCGTCAGAGTTG	qPCR
	*rbm34*-R	GTCACTCGGTCCACTCGGATGT	qPCR
*tep1*	*tep1*-F	GGAATGTGAGAAGGAGGAGGAGAAGA	qPCR
	*tep1*-R	GGCTGAGATGACGGTGCTGTTG	qPCR
*bcl9l*	*bcl9l*-F	TTCTCTGGAGGACAGGTGGAAGG	qPCR
	*bcl9l*-R	GATGAGGAGGAGGCACTGAAGGA	qPCR

### 2.5 Statistical Analysis

Data were expressed as the mean ± standard error (SE). All statistical tests were performed using Statistical Package for the Social Sciences (SPSS) 19.0 (SPSS, Chicago, IL, USA). Significant differences in the data among groups were tested by one-way analysis of variance (ANOVA), followed by Duncan’s *post-hoc* test. The probability level lower than 0.05 (*P* < 0.05) indicated significance difference and lower than 0.01 (*P* < 0.01) is extremely significance.

## 3 Results

### 3.1 Effects of sbGnRH Peptides on Liver Physiological and Biochemical Indexes *In Vivo*

**Figure 1:**
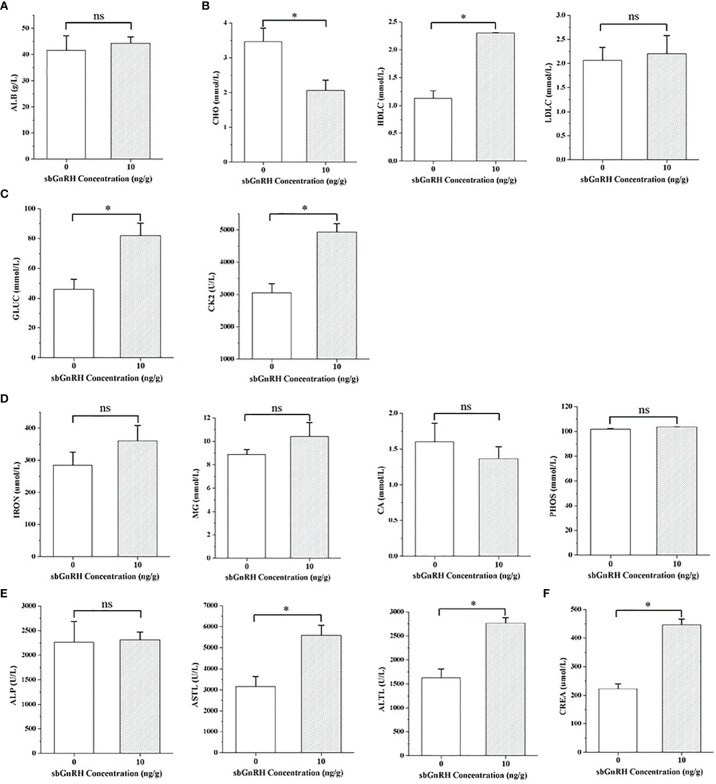
Comparative evaluation of physiological and biochemical indexes in the liver of pompano. **(A)** protein metabolism; **(B)** lipid metabolism; **(C)** energy metabolism; **(D)** inorganic component; **(E)** liver function; **(F)** renal function. Data are expressed as mean ± standard error (SE) (n = 3), and the statistical significance (compared with the control group) was calculated using one-way analysis of variance (ANOVA), followed by Duncan’s *post hoc* test. * indicate statistical differences at *P* < 0.05, respectively. ns, not significant (*P* > 0.05).

The indexes of protein metabolism showed that there was no significant difference in the content of liver ALB in pompano injected with different concentrations of sbGnRH peptides (*P* > 0.05). Compared with the control group, the content of ALB in the treatment group increased (**Figure 1A**). In lipid metabolism, the content of CHO in the treatment group decreased significantly (*P* < 0.05), the content of HDLC increased significantly (*P* < 0.05), and the content of LDLC increased, but there was no significant difference between the treatment group and the control group (*P* > 0.05) (**Figure 1B**). In terms of energy metabolism, the contents of GLUC and CK in the treatment group increased significantly (*P* < 0.05) (**Figure 1C**). There was no significant difference in the contents of IRON, MG, CA and PHOS in the inorganic components (*P* > 0.05), but the contents of IRON, MG and PHOS increased and CA decreased in the treatment group (**Figure 1D**). The content of ALP in the liver function index increased, which had no significant difference with the control group (*P* > 0.05); The contents of AST and ALT were significantly higher than those in the control group (*P* < 0.05) (**Figure 1E**). The content of CREA in the renal function index increased significantly compared with the control group (*P* < 0.05) (**Figure 1F**).

### 3.2 Effects of sbGnRH Peptides on Liver Digestive and Metabolic Enzyme Activities *In Vivo*

**Figure 2:**
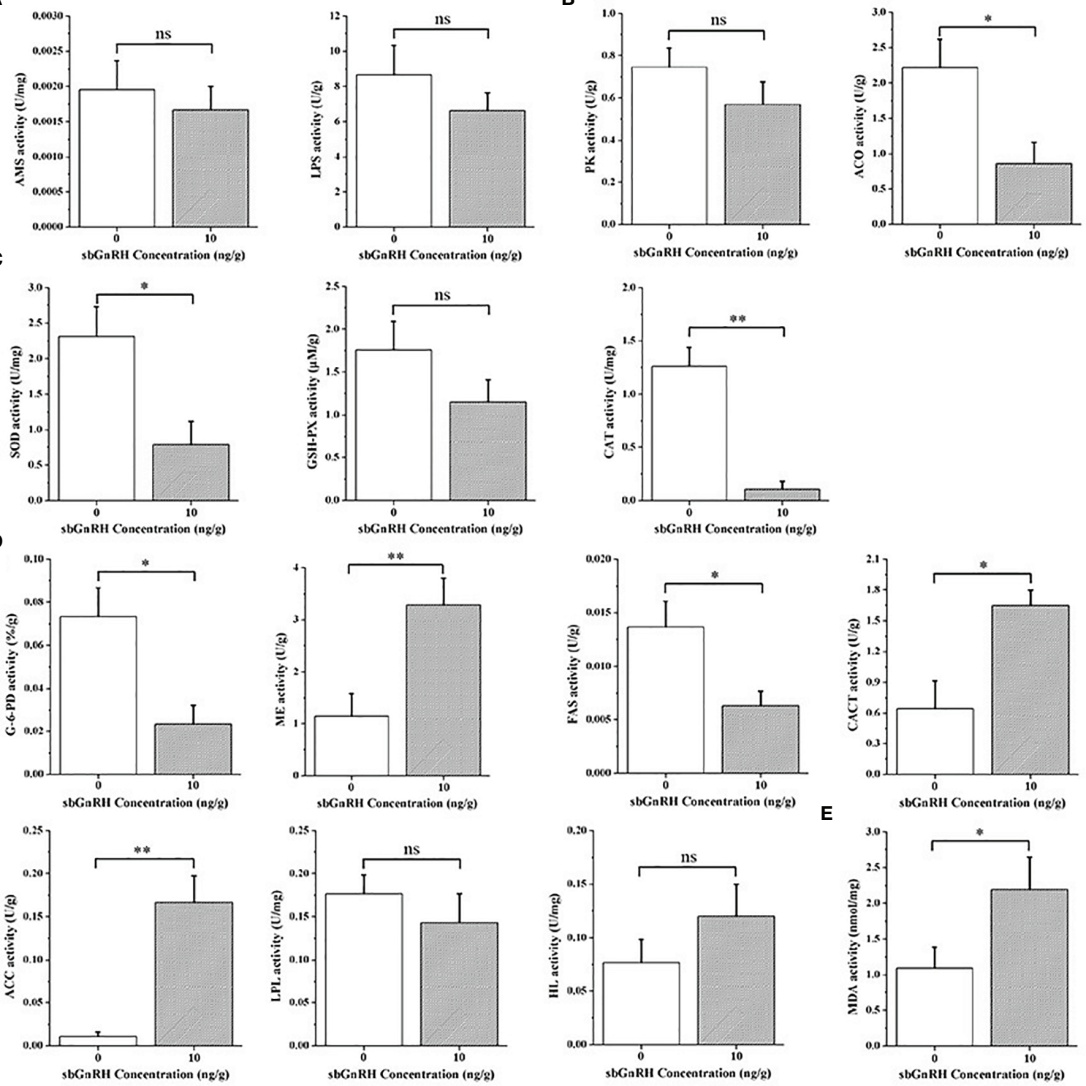
Comparative evaluation of related liver enzyme activities between control group and treatment group. **(A)** digestion; **(B)** carbohydrate metabolism-related enzymes; **(C)** antioxidant defense; **(D)** lipid metabolism; **(E)** Membrane lipid peroxidation. Data are expressed as mean ± standard error (SEM) (n = 3), and the statistical significance (compared with the control group) was calculated using one-way analysis of variance (ANOVA), followed by Duncan’s *post hoc* test. * and ** indicate statistical differences at *P* < 0.05 and *P* < 0.01, respectively. ns, not significant (*P* > 0.05).

After injection of sbGnRH peptides at the concentration of 10 ng/gbw for 3 h, the comparison results of digestive enzyme activities showed that the activities of AMS and LPS in the liver decreased, and there was no significant difference with the control group (*P* > 0.05) (**Figure 2A**). In terms of enzymes related to carbohydrate metabolism, the activities of PK and ACO decreased, but there was a significant difference in ACO compared with the control group (*P* < 0.05) (**Figure 2B**). In terms of antioxidant defense, GSH-PX activity decreased, and there was no significant difference with the control group (*P* > 0.05); SOD (*P* < 0.05) and CAT (*P* < 0.01) activities decreased significantly (**Figure 2C**). In terms of lipid metabolism, LPL activity decreased (*P* > 0.05), G-6-PD and FAS activities decreased significantly compared with the control group (*P* < 0.05), while HL activity increased (*P* > 0.05), CACT (*P* < 0.05), ME and ACC (*P* < 0.01) activities increased significantly compared with the control group (**Figure 2D**). In addition, a significant increase in MDA content reflecting oil rancidity was observed (*P* < 0.05) (**Figure 2E**).

### 3.3 Sequencing and Assembly Results of the Liver Transcriptome of Pompano

Based on Sequencing by Synthesis (SBS) technology, the liver tissue of pompano was sequenced 3 hours after *in vivo* injection of 10 ng/gbw (treatment group) sbGnRH peptides using Illumina hiseq high-throughput sequencing platform. Raw reads were obtained from C-1, C-2, C-3, T-1, T-2, and T-3. After quality control and removal of some low-quality data, clean reads of 21,283,647、19,427,359、21,873,990、21,732,174、23,660,062 and 21,592,338 were obtained respectively. The sequencing quality results show that the Q20 value (percentage of sequences with a sequencing error rate less than 1%) of each group of samples exceeds 97%, the Q30 value (percentage of sequences with a sequencing error rate less than 0.1%) exceeds 92%, and the GC content was about 46.92% - 47.80% (**Table 2**).

**Table 2:** Summary of transcriptome sequencing data of liver in pompano.

Sample	Clean Reads	Clean ReadsQ20 (%)	Clean ReadsQ30 (%)	GC Content (%)
C-1	21,283,647	97.88	94.02	46.92
C-2	19,427,359	98.00	94.29	47.60
C-3	21,873,990	97.66	93.50	47.70
T-1	21,732,174	97.33	92.95	47.36
T-2	23,660,062	98.22	94.78	47.80
T-3	21,592,338	98.05	94.40	47.39

### 3.4 Gene Function Annotation

UniGene sequences were compared with COG, GO, KEGG, KOG, Pfam, Swiss-Prot, eggNOG and NR databases to obtain the annotation information of UniGene (**Table 3**). 25,715 Unigene with annotation information were obtained. Among the results of all database alignment genes, 4527 (17.60%) were annotated in the COG database, 22280 (86.64%) were annotated in the GO database, 20570 (79.99%) were annotated in the KEGG database, 15706 (61.08%) in KOG database, 17050 (66.30%) in Pfam database, 11992 (46.63%) in Swiss-Prot database, 21702 (84.39%) in eggNOG database and 24797 (96.43%) in NR database.

**Table 3:** Statistics of database annotation information.

Database	COG	GO	KEGG	KOG	Pfam	Swiss-Prot	eggNOG	NR
Numbers	4527	22280	20570	15706	17050	11992	21702	24797
Ratio	17.60%	86.64%	79.99%	61.08%	66.30%	46.63%	84.39%	96.43%

**Figure 3:**
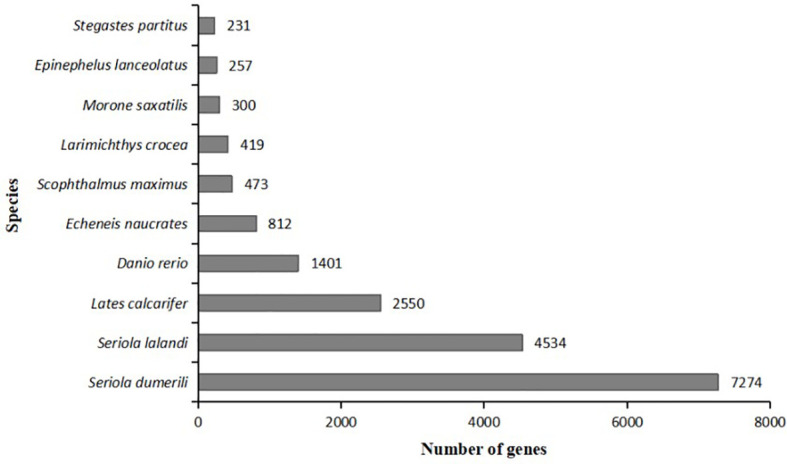
Comparative distribution of NR homologous species. The Y-coordinate represents species; the X-coordinate represents the number of genes.

UniGene sequences were aligned into the NR database. The species with the largest number were *Seriola dumerili* (7274, 29.33%), followed by *Seriola lalandi* (4534, 18.28%), *Lates calcifer* (2550, 10.28%), *Danio rerio* (1401, 5.65%), *Echeneis naurates* (812, 3.27%), *Scophthalmus maximus* (473, 1.91%), *Larimichthys crocea* (419, 1.69%), *Morone saxatilis* (300, 1.21%), *Epilephelus lanceolatus* (257, 1.04%), *Stegastes partitus* (231, 0.93%). This indicates that the species with the closest genetic relationship compared with the liver transcriptome sequencing of pompano is *Seriola dumerili* (**Figure 3**).

### 3.5 Analysis of Differentially Expressed Genes

**Figure 4:**
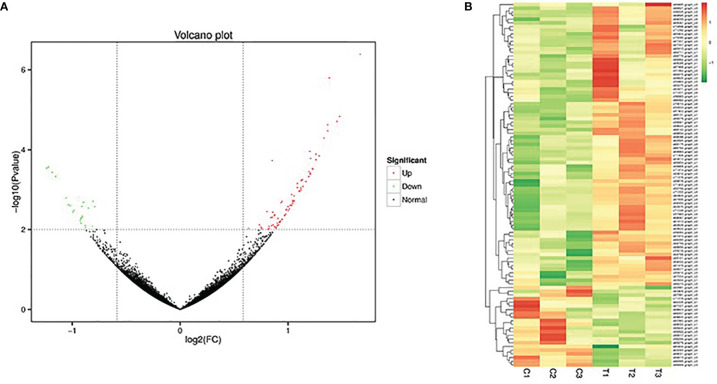
Differential gene expression in the liver of pompano between control group and treatment group. **(A)** Volcano plot of the differences in gene expression. Each dot represents a gene. The green and red dots in the figure represent genes with significant expression differences, green represents down-regulation of gene expression, red represents up-regulation of gene expression, and black dots represent genes with no significant expression differences. **(B)** Heatmap of the hierarchical cluster of DEGs for illustrating the overall pattern of gene expression among different liver samples.

In this study, 99 genes were screened and identified as differentially expressed genes, including 77 up-regulated genes and 22 down-regulated genes (**Figure 4A**). Hierarchical cluster analysis was performed on the screened differentially expressed genes. The results showed that the biological repeat individuals of liver tissue after treatment with different concentrations of sbGnRH peptides could gather respectively, indicating that the repeatability of the sample was more reliable (**Figure 4B**). Based on the expression amount of genes in different samples, the identified differentially expressed genes were annotated. The number of differentially expressed genes annotated was 88, including 21 in COG database, 74 in GO database, 74 in KEGG database, 50 in KOG database, 72 in Pfam database and 56 in Swiss-Prot database, eggNOG database annotation to 76, NR database annotation to 86 (**Table 4**).

**Table 4:** Number statistics of differentially expressed genes annotated.

DEG Set	Annotated	COG	GO	KEGG	KOG	Pfam	Swiss-Prot	eggNOG	NR
Numbers	88	21	74	74	50	72	56	76	86

### 3.6 The Enriched GO Terms and KEGG Pathways

**Figure 5:**
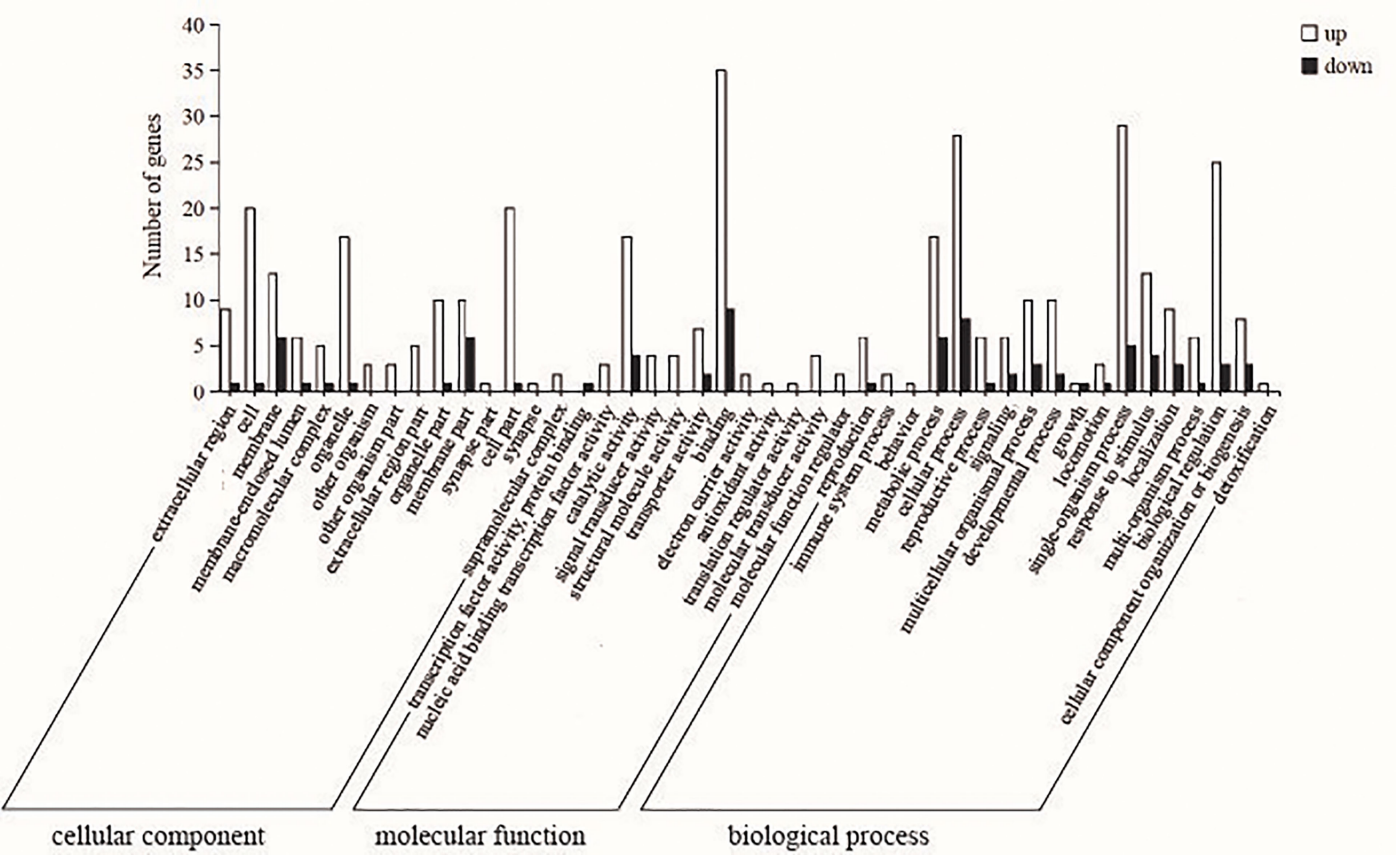
Enrichment analysis of liver differentially expressed genes (DEGs) gene ontology (GO) between control and sbGnRH injection groups. White bars indicate up-regulated genes; Black bars indicate down-regulated genes; the Y-coordinate represents the number of genes; the X-coordinate represents the name of the pathway.

According to GO enrichment analysis, DEGs are divided into three main functional categories: cellular component (CC), molecular function (MF) and biological process (BP), including 15, 12 and 18 subcategories respectively (**Figure 5**). Among them, the DEGs in the CC category were significantly enriched in cell (GO: 0005623), cell part (GO: 0044464) and organelle (GO: 0043226) GO terms. In the MF category, the DEGs were significantly enriched in catalytic activity (GO: 0003824) and binding (GO: 0005488) GO terms. The majority of DEGs in the BP category were associated with cellular processes (GO: 0009987), single-organism process (GO: 0044699) and biological regulation (GO: 0065007) GO terms.

**Figure 6:**
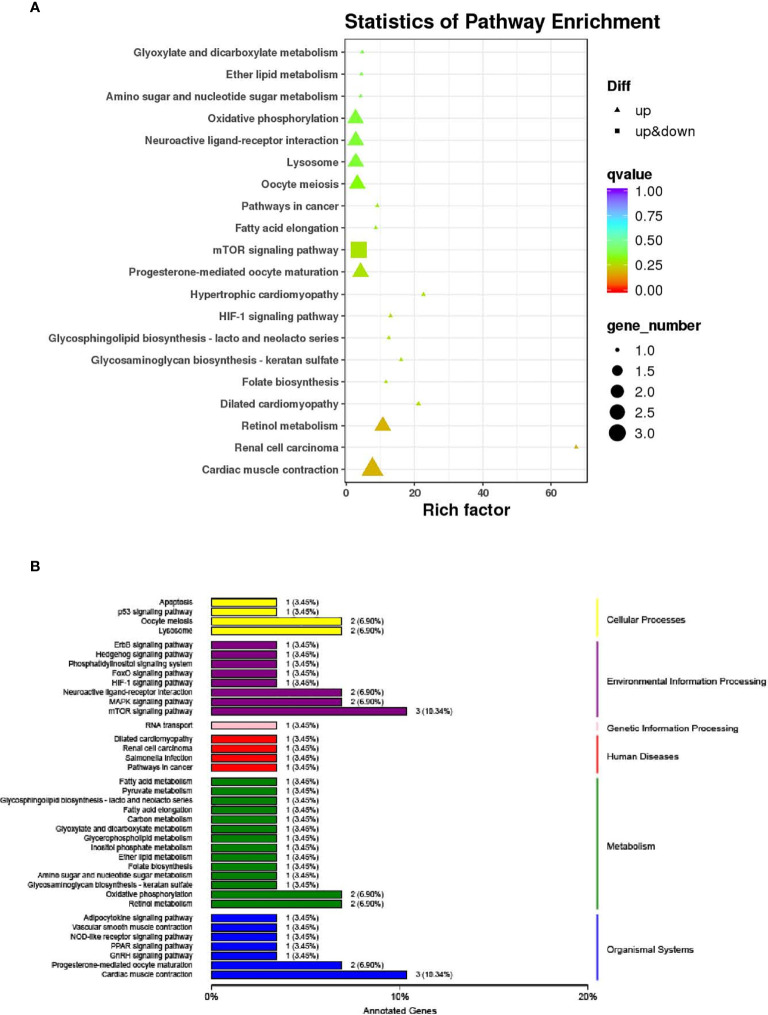
The top 20 significantly enriched KEGG pathways of differentially expressed genes (DEGs). **(A)** The pathways and rich factor are shown in the vertical and the horizontal axis, respectively. The dot size indicates the number of genes and the color indicates the q-value. **(B)** Classification of differentially expressed genes KEGG. The vertical axis is the name of KEGG metabolic pathway, the left part is the specific pathway name, and the right part is the classification category corresponding to each pathway. And the horizontal axis is the Annotated Genes. The number on the column is the number of differentially expressed genes related to this pathway. The same column color represents the same category.

The KEGG pathway enrichment analysis results of liver DEGs show that 41 pathways are enriched, of which the first 20 pathways with the most reliable enrichment significance (the smallest q-value) are shown in **Figure 6A**. These pathways and the typical genes involved can be divided into cellular processes, environmental information processing, genetic information processing, diseases, metabolism and organismal systems (**Figure 6B**). Among them, the following KEGG pathways related to disease immunity were significantly enriched: Dilated cardiomyopathy, Renal cell carcinoma, Salmonella infection and Pathways in cancer; the metabolism related KEGG pathway is significantly enriched: Fatty acid metabolism, Pyruvate metabolism, Glycosphingolipid biosynthesis - lacto and neolacto series, Fatty acid elongation, Carbon metabolism, Glyoxylate and dicarboxylate metabolism, Glycerophospholipid metabolism, Inositol phosphate metabolism, Ether lipid metabolism, Folate biosynthesis, Amino sugar and nucleotide sugar metabolism, Glycosaminoglycan biosynthesis - keratan sulfate, Oxidative phosphorylation and Retinol metabolism (**Figure 6B**). These functional classifications provide a theoretical basis for revealing the effects of sbGnRH on liver diseases, immune and metabolic functions of pompano.

### 3.7 Validation of RNA-Seq Data With qRT-PCR

**Figure 7:**
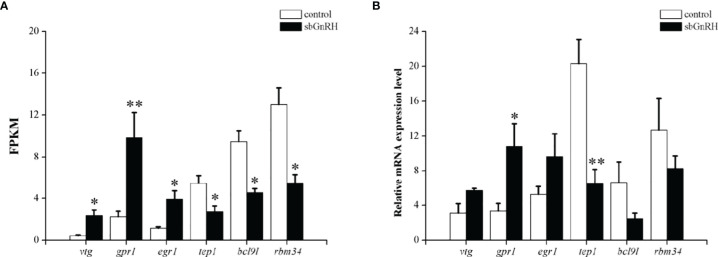
Expression of the gene in the liver of pompano in control and sbGnRH injection groups. **(A)** Transcriptome data (n=3) and **(B)** quantitative polymerase chain reaction (qPCR) results (n=3). Data are presented as mean ± standard error (SEM). * and ** indicate statistical differences at *P* < 0.05 and *P* < 0.01, respectively. The statistical significance (compared with the control group) was calculated using one-way analysis of variance (ANOVA), followed by Duncan’s *post hoc* test.

In this experiment, six genes (*vtg*, *gpr1*, *egr1*, *tep1*, *bcl9l* and *rbm34*) were randomly screened for qPCR verification, in order to verify the accuracy of transcriptome sequencing results. The results showed that the expression of *vtg*, *gpr1* and *egr1* genes was up-regulated and the expression of *tep1*, *bcl9l* and *rbm34* genes was down-regulated after sbGnRH peptides treatment. The qPCR results were consistent with the sequencing results, indicating the accuracy and specificity of transcriptome analysis (**Figure 7**).

## 4 Discussion

Fish biochemical indexes can judge adaptation and nutritional information (37). The protein content in fish is related to metabolism, energy consumption and immune strength. When subjected to external stress, metabolism is strengthened and energy consumption is accelerated, resulting in the decrease of protein content and immune ability (38). After the appropriate concentration of sbGnRH peptides was injected *in vivo*, the ALB content in the liver of pompano increased, and there was no significant difference between the groups. The increase of ALB content may affect the enhancement of the immune level. The relative stability of total protein content is the performance of self-regulation of fish body, which is also related to the experiment, This is similar to the results of *Epinephelus coioides*, *Ctenopharyngodon idella*, *Cyprinus carpio* and *Mylopharyngodon piceus* (39, 40). Triglyceride, cholesterol, low-density lipoprotein and high-density lipoprotein are the four commonly used blood lipid indexes. Any of the first three exceeding the standard belongs to hyperlipidemia, but the low content indicates malnutrition and slow growth, the results of these indices of pompano are consistent with those of *Acipenser schrencki* and *Epinephelus coioides* (41). Combined with transcriptome analysis, the significant increase of HDLC may be related to fatty acid metabolism and glycerol phospholipid metabolism. The expression of ZGC: 55413 protein gene and uncharacterized protein loc791752 isoform X1 gene involved in these metabolisms are up-regulated. Constant glucose concentration plays an important role in maintaining the normal life activities of fish. Usually, glycogen is the core energy storage, and the mode of glycogen utilization of fish carbohydrates is located in the liver and muscle, (42). Among the indicators of energy metabolism, the contents of GLUC and CK increased significantly, which may be related to the beta-1,4-galactosyltransferase 4 gene in glycosaminoglycan biosynthesis and glycosphingolipid biosynthesis and the malate synthase gene in carbon metabolism. After injection of sbGnRH peptides, its expression was up-regulated. AST and ALT are important aminotransferases, which are widely distributed in the cell membrane, cytoplasm and mitochondria. They are often used as factors to evaluate hepatopancreatic function (43). Under normal conditions, the activities of both enzymes are not high, but when tissue cells, especially liver and heart cells, are damaged, the activities of these enzymes increase significantly. Therefore, AST and ALT activities can reflect the damage of fish physiological function (44). The results showed that the contents of AST and ALT increased after injection of 10 ng/gbw sbGnRH peptides, suggesting that sbGnRH may be involved in the immune regulation of liver disease. Creatinine is mainly the final product of creatine and creatine phosphate metabolism and an indicator of gill and renal function. Therefore, creatinine content is closely related to muscle activity (45, 46). Creatinine excretion can reflect the function of the kidney. When kidney disease occurs, creatinine excretion is blocked and the content of creatinine in the blood increases significantly (47).

sbGnRH is widely distributed in the nervous, endocrine, reproductive, digestive and immune systems. By transmitting information, all systems are coordinated and unified, and sbGnRH in different tissues has different biological functions (9). Aquatic animals obtain nutrition and energy almost entirely through feeding, digestion and absorption. The absorption and utilization of nutrients strongly depend on the activity of digestive enzymes and make a positive contribution to the growth ability of fish (48). In this study, ME activity and ACC activity in lipid metabolizing enzymes were enhanced, further indicating that sbGnRH may participate in pyruvate metabolism, glyoxylate and dicarboxylate metabolism, resulting in the up regulation of malate synthase gene expression. It has been shown that the higher growth performance of male tilapia (*Oreochromis niloticus*) may be due to its strong ability to digest and metabolize nutrients (49). It has been well confirmed that exogenous steroid treatment has different effects on energy distribution patterns. For example, the change of androgen level in the reproductive season is related to the change of energy distribution (50); Elevated plasma testosterone levels cause changes in liver metabolism, which may be related to the process of energy redistribution (51). Combined with the metabolic enzyme activity index and liver transcriptome analysis of pompano, sbGnRH may be involved in non-reproductive processes such asdigestion, lipid metabolism and antioxidant defense of pompano. However, the mechanism of GnRH in the digestive system and immune system is still a relatively unexplored research field. Our preliminary findings will pave the way for a more comprehensive understanding of this complex action system.

RNA-seq is an important method for quantitative transcriptional expression to clarify the response of environmental factors such as salinity, temperature, pH, dissolved oxygen and sex hormones in species or organisms (52–56). In this study, the mRNA function of liver response to sbGnRH injection was studied by detecting the liver RNA-seq of pompano injected with 10 ng/gbw concentrations of sbGnRH peptides. The changes of DEGs in the liver of sbGnRH injection group and control group were analyzed by transcriptome and qPCR. Three hours after injection of 10 ng/gbw sbGnRH peptides, we identified DEGs in pompano and detected its expression pattern. 99 DEGs were identified in the control group and sbGnRH treatment group. In this study, GO enrichment analysis showed that after injection of sbGnRH peptides into pompano, DEGs were significantly enriched in cells, cell parts, binding, metabolic processes, cellular processes and biological regulation. In teleosts, it has been identified to be related to the regulation of GnRH (57–59). The results of this study show that KEGG pathway related to disease immunity and metabolism is significantly enriched, suggesting that sbGnRH plays a non-reproductive related function in different tissues (60–62). In this study, sbGnRH injection significantly up-regulated genes related to reproductive regulation,early growth response protein 1, cytoplasmic polyadenylation element-binding protein 1 and ribosomal protein S6 kinase alpha-3 isoform X2. sbGnRH is involved in the regulation of oocyte meiosis, progesterone mediated oocyte maturation and GnRH signaling pathway. Therefore, it is necessary to further study the reproductive and non-reproductive functions of sbGnRH in pompano.

## 5 Conclusions

In this study, transcriptome sequencing technology was used as a tool to explore the function of sbGnRH in regulating physiological and biochemical indexes and metabolic enzyme activities in the liver of pompano. The results show that sbGnRH is involved in the immune regulation of liver disease and the regulation of digestive and metabolic enzyme activities, suggesting that sbGnRH has non reproductive related functions. sbGnRH may play different biological functions in the immune system and digestive system, to maintain the homeostasis of the body. Therefore, we know that GnRH has many functions and can regulate many physiological processes. It not only plays an important role in the reproductive system, but also regulates the non-reproductive system. The data of this study are of great significance to further clarify the non-reproductive related function of sbGnRH.

## Data Availability Statement

The original contributions presented in the study are publicly available. This data can be found here: NCBI: SRX14408583, SRX14408582, SRX14408581, SRX14408580, SRX14408579,SRX14408578.

## Ethics Statement

The animal study was reviewed and approved by the Animal Research and Ethics Committees of Fisheries College of Guangdong Ocean University. Written informed consent was obtained from the owners for the participation of their animals in this study.

## Author Contributions

XR: Investigation, Data curation, Formal analysis, Writing–original draft. JL: Data curation, Formal analysis, Resources. XL: Data curation. CN: Investigation. YG: Investigation, Methodology. GL: Investigation. HC: Data curation, Funding acquisition, Methodology, Resources, Supervision, Writing– original draft, Writing - re-view and editing. All authors contributed to the article and approved the submitted version.

## Funding

This article was supported by grants from the Key Research and Development Program of Guangdong (2021B202020002), National Natural Science Foundation of China (41706174), The talent team tender grant of Zhanjiang marine equipment and biology (2021E05035), the Guangdong Basic and Applied Basic Research Foundation (2019A1515010958 and 2019A1515012042), the Southern Marine Science and Engineering Guangdong Laboratory (Zhanjiang) (ZJW-2019-06).

## Conflict of Interest

Author JL is employed by Guangdong Havwii agricultural Group Co., LTD.

The remaining authors declare that the research was conducted in the absence of any commercial or financial relationships that could be construed as a potential conflict of interest.

## Publisher’s Note

All claims expressed in this article are solely those of the authors and do not necessarily represent those of their affiliated organizations, or those of the publisher, the editors and the reviewers. Any product that may be evaluated in this article, or claim that may be made by its manufacturer, is not guaranteed or endorsed by the publisher.
