# Supplementary material for: Physiological Effects and Transcriptomic Analysis of sbGnRH on the Liver in Pompano (Trachinotus ovatus)
Source: Front Endocrinol (Lausanne). 2022 May 2;13:869021. doi: 10.3389/fendo.2022.869021 (PMC9108241; doi:10.3389/fendo.2022.869021)
Supplement: Supplementary file 1 [file Table_1.docx]

**Supplementary File**

**Table S1:** The product numbers of commercial assay kits.

| **Enzyme** | **Kit No.** | **Manufacturer** |
| --- | --- | --- |
| Amylase (AMS) | C016-1-1, C016-2-1 | Nanjing Jiancheng Bioengineering Institute |
| Glucose-6-phosphate dehydrogenase (G-6-PD) | A027-1-1 | Nanjing Jiancheng Bioengineering Institute |
| Malic enzyme (ME) | BC1125 | Solarbio Life Sciences |
| pyruvate kinase (PK) | A076-1-1 | Nanjing Jiancheng Bioengineering Institute |
| Acyl-CoA oxidase (ACO) | H232 | Nanjing Jiancheng Bioengineering Institute |
| Lipase (LPS) | E1019 | Applygen Technologies Inc. |
| Fatty acid synthase (FAS) | H231 | Nanjing Jiancheng Bioengineering Institute |
| Carnitine-acylcarnitine translocase (CACT) | SEB657Ra | Wuhan Cloud-Clone Corp. |
| Acetyl-CoA carboxylase (ACC) | H232 | Nanjing Jiancheng Bioengineering Institute |
| Lipoprotein lipase (LPL) | A067-1-1 | Nanjing Jiancheng Bioengineering Institute |
| Superoxide dismutase (SOD) | A001-3-1 | Nanjing Jiancheng Bioengineering Institute |
| Phospholipid hydroperoxide glutathione peroxidase (GSH-PX) | A005-1-1 | Nanjing Jiancheng Bioengineering Institute |
| Catalase (CAT) | A007-1-1 | Nanjing Jiancheng Bioengineering Institute |
| malondialdehyde (MDA) | [A003-1-1](http://www.njjcbio.com/products.asp?id=288) | Nanjing Jiancheng Bioengineering Institute |
